# HPV11 targeting PPARA regulates the autophagy to inhibit the occurrence and development of nasal inverted papilloma

**DOI:** 10.3389/fonc.2025.1743808

**Published:** 2026-01-28

**Authors:** Gaohan Zhu, Liying Zheng, Weining He, Xiaoping Chen, Shuixian Huang, Mingming Jin, Yi Zhang

**Affiliations:** 1 School of Gongli Hospital Medical Technology, University of Shanghai for Science and Technology, Shanghai, China; 2 Department of Otorhinolaryngology Head and Neck Surgery, Pudong Gongli Hospital, Shanghai University of Medicine and Health Sciences, Shanghai, China; 3 Postgraduate Training Base at Shanghai Gongli Hospital, Ningxia Medical University, Shanghai, China; 4 Department of General Practice, Caolu Community Health Service Center, Pudong New Area, Shanghai, China; 5 Shanghai Key Laboratory of Molecular Imaging, Shanghai University of Medicine and Health Sciences, Shanghai, China

**Keywords:** autophagy, autophagy inhibition, HPV11, nasal inverted papilloma, PPARA

## Abstract

**Introduction:**

Nasal inverted papilloma (NIP) is closely associated with human papillomavirus (HPV) infection, with HPV11 showing the highest expression levels in NIP tissues. However, the mechanism of its genomic integration remains incompletely understood. This study investigated frequent viral integration at the peroxisome proliferator-activated receptor alpha (PPARA) gene locus—a known regulator of autophagy—and its potential role in HPV11-mediated pathogenesis.

**Methods:**

High-throughput sequencing of HPV-positive specimens identified PPARA as a common integration site. HPV11 E6/E7 overexpression, PPARA overexpression, and PPARA knockdown models were established in human nasal epithelial cells (HNEpC). Cell proliferation, migration, and autophagy levels were assessed. The role of PPARA in proliferation and autophagy modulation was further validated using a nude mouse xenograft model. Additionally, the autophagy inhibitor 3-Methyladenine (3-MA) was applied to evaluate its effects on proliferation and migration.

**Results:**

HPV11 E6/E7 overexpression significantly enhanced cell proliferation and migration. In contrast, PPARA overexpression promoted autophagy and suppressed proliferation and migration. Inhibition of autophagy by 3-MA reversed the suppressive effects mediated by PPARA. *In vivo* experiments confirmed the proliferative role of HPV11 E6/E7 and the autophagy-modulating function of PPARA.

**Discussion:**

HPV11 exerts dual effects on nasal mucosal cells: promoting proliferation and migration via E6/E7, while concurrently inducing an inhibitory effect through PPARA-mediated autophagy activation. The suppression of autophagy reversed the PPARA-driven inhibition, indicating a key role for the autophagy pathway. These findings suggest that PPARA targeting may be crucial in the pathogenesis of NIP, highlighting a complex interaction between HPV11 integration and host autophagy regulation.

## Introduction

Nasal intraepithelial papilloma (NIP) was first described in 1854 (1). Predominantly affecting middle-aged males, it represents the most common benign epithelial tumor of the nasal cavity and paranasal sinuses, accounting for approximately 0.5% to 4.0% of all nasal cavity tumors. Histologically, NIP is characterized by proliferating epithelium growing in a branching, inverted pattern into the underlying stroma, forming solid or trabecular epithelial nests and crypts of varying sizes, often resulting in substantial tissue destruction. Surgical excision remains the primary and most effective treatment modality. However, NIP is classified as a borderline tumor due to its local invasiveness, malignant transformation and high recurrence rate (10%-25.3%) after surgical resection (2, 3). Triggering factors for recurrence or malignant conversion include viral infections, chronic inflammation, and environmental exposures. Notably, both domestic and international studies suggest viral infection may represent one of the initiating factors in the development and progression of NIP.

Human papillomavirus (HPV) is a double-stranded DNA virus possessing oncogenic potential. Based on this carcinogenic capacity, over 200 subtypes are categorized as high-risk or low-risk types (4, 5). Low-risk subtypes, such as those including HPV11, are typically associated with benign epithelial hyperplasia (e.g., papillomas). A key carcinogenic mechanism involves the sustained expression of viral E6 and E7 proteins following viral integration into the host genome, which disrupts cell cycle control and promotes cellular proliferation (6). Although HPV, particularly low-risk types, is strongly implicated in the development of nasal inverted papilloma (NIP), the precise molecular mechanisms driving this process remain unclear.

Our research group has identified a detection rate of 64.36% for HPV in NIP tissue, predominantly low-risk HPV11. Clarifying the distribution of HPV subtypes within NIP may aid in predicting clinical behavior and tumor progression (7). To further investigate its role in NIP, we first screened 37 HPV subtypes in NIP using the HybriMax hybridization array technology, followed by high-throughput sequencing of eight HPV-positive and two HPV-negative samples. This study identified six high-frequency HPV integration sites, among which the nuclear receptor gene PPARA was confirmed as a stable integration site potentially crucial for the development and progression of NIP.

Peroxisome proliferator-activated receptor alpha (PPARA) was first identified in the early 1990s. It serves as a primary regulator of hepatic lipid metabolism and also participates in modulating glucose metabolism, inflammation, and cell proliferation (8). Accumulating evidence indicates that PPARA plays a central role in maintaining cellular homeostasis through multiple mechanisms, including direct transcriptional activation of autophagy-related genes (9), upregulation of TFEB (10, 11), and regulation of selective autophagy such as lipophagy (12). Consequently, targeting PPARA has emerged as a promising strategy for modulating autophagy in various diseases.

Autophagy constitutes a conserved cellular clearance pathway, exerting context-dependent roles in epithelial homeostasis and pathogenesis. Within specific contexts of benign epithelial proliferative disorders such as papillomatosis, autophagy is hypothesized to influence key disease-driving processes. These may include regulating the transformation and differentiation states of epithelial cells undergoing viral infection or hyperproliferation, aiding cellular adaptation to the local inflammatory microenvironment—all central to papillomatosis formation and progression (4). Importantly, Zi et al. (13) demonstrated that enhanced autophagic activity is directly observable in clinical NIP specimens, underscoring autophagy’s specific relevance to papilloma biology.

Despite these insights, the mechanism by which PPARA influences HPV-positive NIP and whether it modulates the tumor microenvironment remains unexplored. Therefore, based on clinical and genomic observations, we investigate whether HPV11 integration into the PPARA locus alters PPARA expression and autophagy regulation in nasal epithelial cells, thereby influencing their proliferation and contributing to the development and progression of NIP. Using molecular, cellular, and animal models, our research aims to elucidate this mechanism, providing a breakthrough in understanding HPV-induced pathogenesis in NIP.

## Materials and methods

### Cell lines and cell culture

#### Cell line selection

Human nasal epithelial cells (HNEpC) were cultured in DMEM/F-12 medium (Base Media, Shanghai, China) supplemented with 10% fetal bovine serum (FBS, BIOEXPLORER, USA) and 1% penicillin/streptomycin (PS, Gibco, USA). The cells were maintained at 37°C in a humidified atmosphere containing 5% CO_2_ and 95% air. When cells reached 70–80% confluence, they were digested with 0.25% trypsin-EDTA solution (Gibco, USA), the reaction was terminated by addition of complete medium, and after centrifugation at 1200 rpm for 4 min, cells were prepared for experiments or resuspended in new medium for subculture.

#### Lentiviral transfection

Cells from each group were seeded into 6-well plates at 2 × 10^5^ cells per well. Following serum-free starvation for 6 hours, cells were transfected at a multiplicity of infection (MOI) of 30 using a viral titer of 1 × 10^8^ TU/mL (GeneChem, Shanghai, China) in the presence of 8 μg/mL Polybrene (Sigma-Aldrich) to enhance efficiency. Control groups received an equal volume of blank control solution (medium containing Polybrene). Solutions were gently mixed and incubated overnight (approximately 16 hours) in a CO_2_ incubator (37°C, 5% CO_2_). Following recovery in fresh complete medium for 24 hours, cells were screened with 1.6 μg/mL puromycin for 72 hours. Transfection efficiency was preliminarily assessed under fluorescence microscopy. Prior to functional analysis, successful genetic manipulation was confirmed at both mRNA and protein levels via RT-qPCR and Western blot, respectively.

#### Cell model construction

In this study, the early gene products E6/E7 of HPV were used to mimic HPV infection in host cells. A lentiviral vector expressing HPV11 E6/E7 was stably transfected into HNEpC to establish an HPV11 lentivirus infection model. This E6/E7-overexpressing model was employed to specifically investigate the tumorigenic functions of these viral proteins in driving cellular phenotypes, rather than to recapitulate the full HPV11 infectious cycle. Subsequently, based on the HPV11 E6/E7-overexpressing HNEpC background, lentiviruses carrying either overexpression or knockdown constructs of PPARA were stably transfected to establish corresponding PPARA modification models. The experimental groups were designed as follows: HNEpC as the negative control, HPV11E6/E7-HNEpC as the positive control, OE-PPARA–HPV11E6/E7–HNEpC as the PPARA overexpression group, and Si-PPARA–HPV11E6/E7–HNEpC as the PPARA knockdown group. HNEpC cells served as the negative control group, stably transfected with HPV11E6/E7 control empty vector lentivirus. HPV11E6/E7-HNEpC cells functioned as the positive control group, stably overexpressing HPV11E6/E7, and were further stably transfected with PPARA control empty vector lentivirus to control for background effects associated with viral transduction.

### Cell proliferation assay

#### Cell count Kit 8 (CCK8) determination

Cell viability was assessed using a CCK-8 assay kit (Yeasen, Shanghai, China) at 1, 2, and 3 days post-infection. Cells were seeded into 96-well plates at a density of 4,000 cells/well, with six replicate wells per group. After allowing the cells to adhere for 5 hours, 10 μL of CCK-8 solution was added to each well at 0, 24, 48, and 72 hours, followed by incubation at 37°C in the dark for 1 hour. Optical Density (OD) at 450 nm was measured by enzyme labeling. Data were analyzed with GraphPad Prism 9.0 software to determine the relative proliferation rate.

#### 5-ethyl-2'-deoxyuridine (EdU) assay

Cell proliferation was evaluated using an EdU assay kit (UElandy, Suzhou, China). Cells were seeded in 24-well plates (4×10^4^ cells/well) and cultured overnight. Then, 500 μL of medium containing 10 μmol/L EdU was added to each well and incubated for 2 hours at 37°C with 5% CO_2_. After incubation, the cells were fixed with 4% paraformaldehyde and permeabilized with 0.5% Triton X-100. EdU staining solution was applied followed by DAPI counterstaining. Images were captured using a fluorescence microscope (Leica, Shanghai, China). The EdU-positive rate was calculated as the ratio of EdU-positive cells (red fluorescence) to Hoechst-positive nuclei (blue fluorescence).

#### Colony formation assay

Cells from the four experimental groups were seeded in 6-well plates at a density of 1,000 cells/well and cultured in DMEM/F12 medium supplemented with 10% FBS for one week at 37°C in a 5% CO_2_ incubator. After incubation, the cells were washed twice with PBS (Servicebio, Wuhan, China), fixed with 4% paraformaldehyde for 20 minutes, and stained with 0.5% crystal violet for 20 minutes. After staining, image acquisition was performed for each well. For colonies containing ≥50 cells, ImageJ software (version 1.54p, National Institutes of Health, USA) was used for counting and semi-automated quantification and was compared with untreated controls.

### Migration assay

#### Wound healing assay

Cells from each group in the logarithmic growth phase were seeded into 6-well plates at a density of 4×10^5^ cells/well. All cultures were maintained in DMEM/F12 medium supplemented with 10% FBS. When cell confluence reaches 90%–95%, pre-treat with 2 μg/mL mitomycin C for 2 hours to inhibit cell proliferation. Subsequently, create a ‘wound’ in the monolayer using the tip of a 200μL pipette. The dislodged cells were gently washed away with PBS, and serum-free medium was added. Images of the scratch were captured immediately (0 hours) and for two consecutive days under an inverted microscope at 100× magnification. The wound area was measured using ImageJ software(version 1.54p, National Institutes of Health, USA), and the percentage of wound closure (migration ability) was calculated as follows: Wound healing rate (%) = [(0-hour wound area - time-point wound area)/0-hour wound area] × 100%.

#### Transwell assay

Cells from each group were resuspended in 200 μL of serum-free medium and seeded into the upper chamber of an 8 μm pore-size Transwell plate at a density of 2×10^4^ cells per well. The lower chamber was filled with 500 μL of medium containing 15% FBS as a chemoattractant. Negative control well: The lower chamber contains only serum-free basal medium. After 24 hours of incubation, the Transwell membrane was fixed with 4% paraformaldehyde for 20 minutes and stained with 0.1% crystal violet for 20 minutes. Under a 100× inverted microscope, three random fields of view per well were selected, and cells migrating through the pores were counted using ImageJ software (version 1.54p, National Institutes of Health, USA). Statistical comparisons were performed on the migration cell counts across each group. The migration cell counts for the experimental groups were adjusted by subtracting the average value from the negative control group.

### *In vivo* experiment

A subcutaneous tumor model was established in nude mice to evaluate the *in vivo* tumorigenicity of genetically modified HNEpC cells. In brief, female BALB/c nude mice were injected with HNEpC cells, HPV11E6/E7-HNEpC cells, or OE-PPARA-HPV11E6/E7-HNEpC cells (2×10^6^ cells/mouse) to form solid tumors. This model directly assesses the impact of PPARA overexpression on HPV11 E6/E7-driven tumor growth. Mice were monitored for body weight and tumor volume every three days for 21 consecutive days. Subsequently, subcutaneous tumor tissue was harvested for immunofluorescence and immunohistochemical analysis. All animal experimental procedures were approved by the Ethics Committee of Shanghai University of Medicine and Health Sciences (Ethics Approval Number: 2024-16-371426200111254828).

### Immunofluorescence staining

#### Cellular immunofluorescence

After sterilization, the coverslips were coated with 0.1 mg/mL polylysine for 10 minutes to enhance cell adhesion. The coated slides were then placed into 6-well plates and seeded with target cells at a density of 4 × 10^5^ cells per well. Cells were cultured at 37 °C under 5% CO_2_ until they reached 70–80% confluence. The culture medium was aspirated and discarded, and the cells were rinsed with PBS followed by fixation with 4% paraformaldehyde at room temperature for 15 minutes.

#### Tissue immunofluorescence

For tissue samples, subcutaneous tumor tissues were fixed in 4% paraformaldehyde, dehydrated, embedded in paraffin, and sectioned. The sections were then dewaxed and rehydrated, after which antigen retrieval was performed.

Both cell samples (on coverslips after fixation) and tissue sections underwent the following routine staining procedure: Endogenous peroxidase activity was blocked, and samples were washed with an immunostaining blocking solution containing Triton X-100. The primary antibody anti-LC3B (1:2000, #3868, Cell Signaling Technology, Boston, USA) was incubated overnight at 4°C. The negative control omitted the primary antibody step, substituting antibody dilution buffer. Subsequently, the corresponding fluorescent secondary antibody was incubated for 1 hour at room temperature in the dark. Following DAPI counterstaining to visualize cell nuclei, autofluorescence quenching was performed before signal detection. Sections were sealed with an anti-fade mounting medium and examined under a fluorescence microscope (Leica DMi8). All samples were imaged at 50× or 100× magnification with consistent exposure times across groups. For LC3B antibody immunofluorescence analysis, three random fields of view per sample were examined.

### Immunohistochemical analysis

Tumor tissue samples were fixed in 10% neutral buffered formalin, embedded in paraffin, and sectioned at a thickness of 5 μm. After dewaxing and rehydration, antigen retrieval was performed by heating the sections in sodium citrate buffer (10 mM, pH 6.0) using a microwave oven. Sections from each group of nude mice were stained with an anti-Ki67 antibody (1:500; #27309-1-AP, Proteintech, Wuhan, China). The stained sections were examined using an AxioPhot light microscope (Carl Zeiss AG, Oberkochen, Germany) equipped with a digital camera to evaluate relevant indices following the different treatments. The proportion of positive cells was quantified using ImageJ software (version 1.54p, National Institutes of Health, USA). and used as the standard for statistical analysis.

### Quantitative real-time polymerase chain reaction

Quantitative real-time PCR (qRT-PCR) was performed to analyze the relative mRNA expression in cells from each fraction. Total RNA was extracted using the RNAfast2000 kit (Fastagen), and complementary DNA (cDNA) was synthesized with a cDNA synthesis kit (Takara, Otsu, Japan). Primer sequences were designed via the PrimerBank website and synthesized by Sangon BioEngineering Co., Ltd. (Shanghai, China). RNA concentration and purity were determined using a NanoDrop spectrophotometer (Thermo Fisher Scientific, Waltham, MA, USA). The primer sequences used are listed in **Table 1**.

**Table 1:** The primers for qRT-PCR.

Gene	Forward primer (from 5’ to 3’)	Reverse primer (from 5’ to 3’)
GAPDH	GAGTCCACTGGCGTCTTCAC	ATGGTTCACACCCATGACGA
HPV11E6/E7	GGAAGGGTCGTTGCTTACACT	TGTCCACCTCATCTTCTGAGC
PPARA	TTCGCAATCCATCGGCGAG	CCACAGGATAAGTCACCGAGG

qRT-PCR amplification was carried out on a StepOnePlus Real-Time PCR System (Thermo Fisher Scientific) using SYBR Green PCR Master Mix (Yeasen, Shanghai, China). The thermocycling protocol consisted of an initial step at 95°C for 30 seconds, followed by 40 cycles of 95°C for 10 seconds and 60°C for 30 seconds. Relative gene expression levels were calculated using the 2^(–ΔΔCt) method, with GAPDH serving as the internal reference gene. Expression values in control samples were set to one for normalization of the target genes.

### Western blot analysis

Total proteins were extracted from transduced cells using RIPA lysis buffer (RIPA Lysis Buffer Strong, Beyotime, China) supplemented with protease inhibitors (Phenylmethanesulfonyl fluoride, Beyotime, China). Protein concentrations were determined with a BCA protein assay kit (Yeasen, Shanghai, China). Proteins (20 μg/lane) were separated by electrophoresis on 10% or 15% SDS-PAGE gels and transferred to PVDF membranes. The membranes were then blocked with 5% skim milk for 1 hour at room temperature. Primary antibody incubation was carried out overnight at 4 °C with gentle shaking using the following antibodies: anti-PPARA (1:3000, #66826-1-Ig, Proteintech, Wuhan, China), anti-HPV11E6 (1:1000, #MBS448108, MyBioSource, San Diego, USA), anti-HPV11E7 (1:1000, #ab100967, Abcam, Shanghai, China), anti-LC3B (1:1000, #T55992, Abmart, Shanghai, China), anti-P62/SQSTM1 (1:10000, #18420-1-AP, Proteintech, Wuhan, China), anti-E-cadherin (1:20000, #20874-1-AP, Proteintech, Wuhan, China), anti-N-cadherin (1:500, #22018-1-AP, Proteintech, Wuhan, China), anti-β-actin (1:20000, #66009-1-Ig, Proteintech, Wuhan, China), and anti-GAPDH (1:50000, #60004-1-Ig, Proteintech, Wuhan, China). GAPDH and β-actin were used as internal controls. The following day, the membranes were incubated for 1 hour with horseradish peroxidase (HRP)-conjugated secondary antibodies: goat anti-mouse IgG (1:500, Proteintech, Wuhan, China) or goat anti-rabbit IgG (1:500, Proteintech, Wuhan, China), with gentle shaking. Protein bands were visualized using an ECL Western Blotting kit (Vazyme, Nanjing, China) and the Tanon 5200 luminescent Imaging system (Tanon, Shanghai, China). Band intensities were quantified using ImageJ software (bundled with Zulu OpenJDK 13.0.6).

### Statistical analyses

All experiments were repeated at least three times. Differences between groups were assessed using one-way analysis of variance (ANOVA), with data expressed as means. A p-value < 0.05 was considered statistically significant. Statistical analysis was performed using GraphPad Prism 9.0 software (GraphPad Software, San Diego, CA, USA).

## Results

### Stable integration of the PPARA gene into the host genome after HPV infection

After identifying HPV integration sites, we annotated these sites using human genome reference data, retaining only those located within specific genomic regions. Subsequently, a pupil map was generated for each patient (see **Supplementary Materials** for details). By integrating all HPV integration analysis location data, a comprehensive map of viral gene integration sites within human chromosomes in HPV-positive samples was constructed. This study is the first to focus on the high-frequency HPV integration site PPARA and its interaction with human chromosomes in NIP tissues.

### PPARA regulates HPV11E6/E7-induced proliferation in HNEpC cells

**Figure 1:**
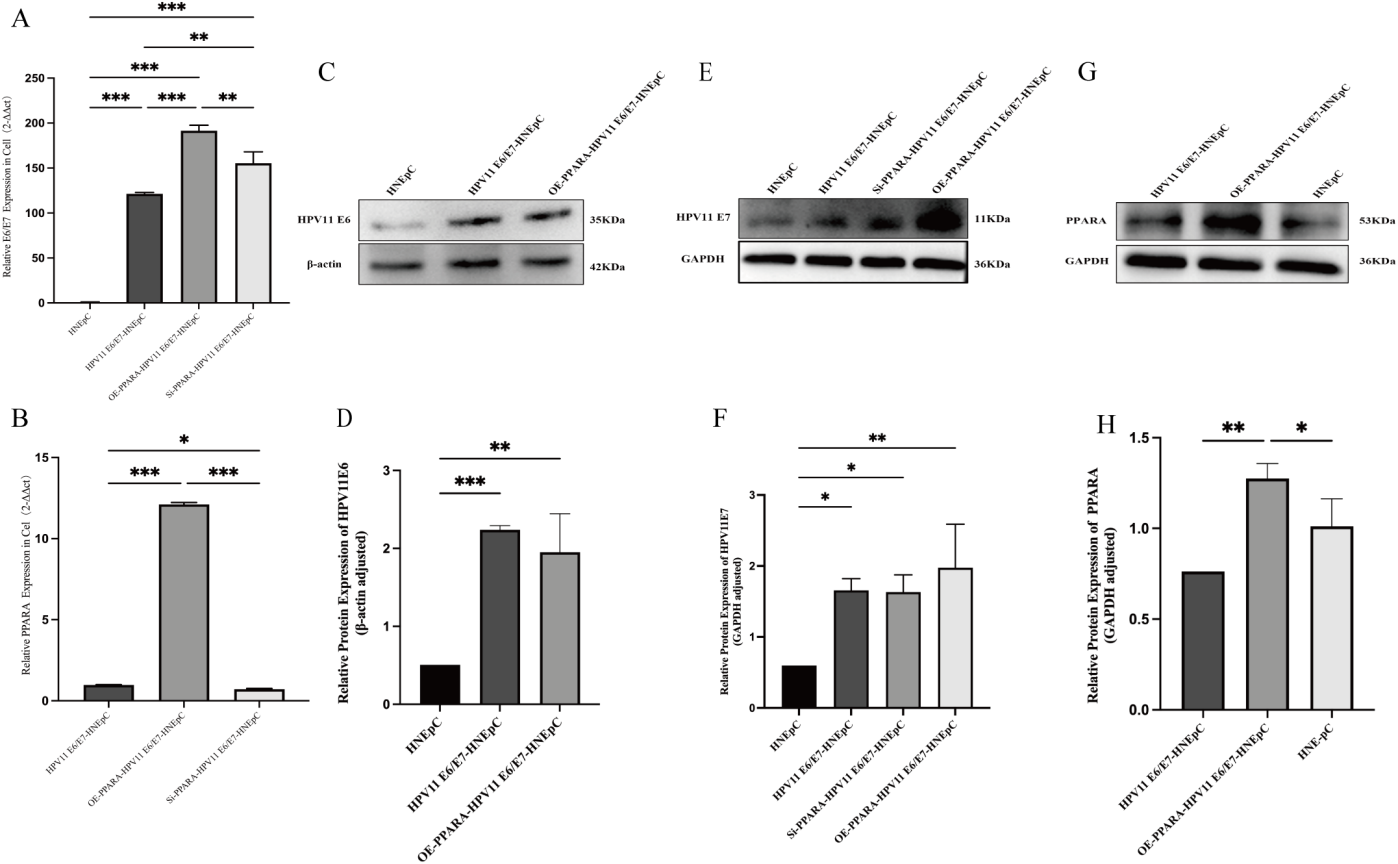
To verify the stable integration of PPARA gene into the host genome after HPV infection **(A, B)** qRT-PCR analysis of HPV11E6/E7 and *PPARA* mRNA expression across groups. **(C–H)** The expression of HPV11E6, HPV11E7 and PPARA in each group was verified by Western blot, and the gray value of each band was analyzed by Image J. Data are presented as mean ± SD; ***P < 0.001, **P < 0.01, *P < 0.05, ns: not significant.

The HPV11E6/E7 overexpression vector was constructed and stably transfected into HNEpC cells to establish an HPV11E6/E7-positive cell model. Subsequently, lentiviruses mediating either overexpression or knockdown of PPARA were stably introduced into the established model. The expression levels of HPV11E6, HPV11E7 and PPARA across all experimental groups were confirmed by qRT-PCR and Western blot analysis (**Figures 1A–H**).

**Figure 2:**
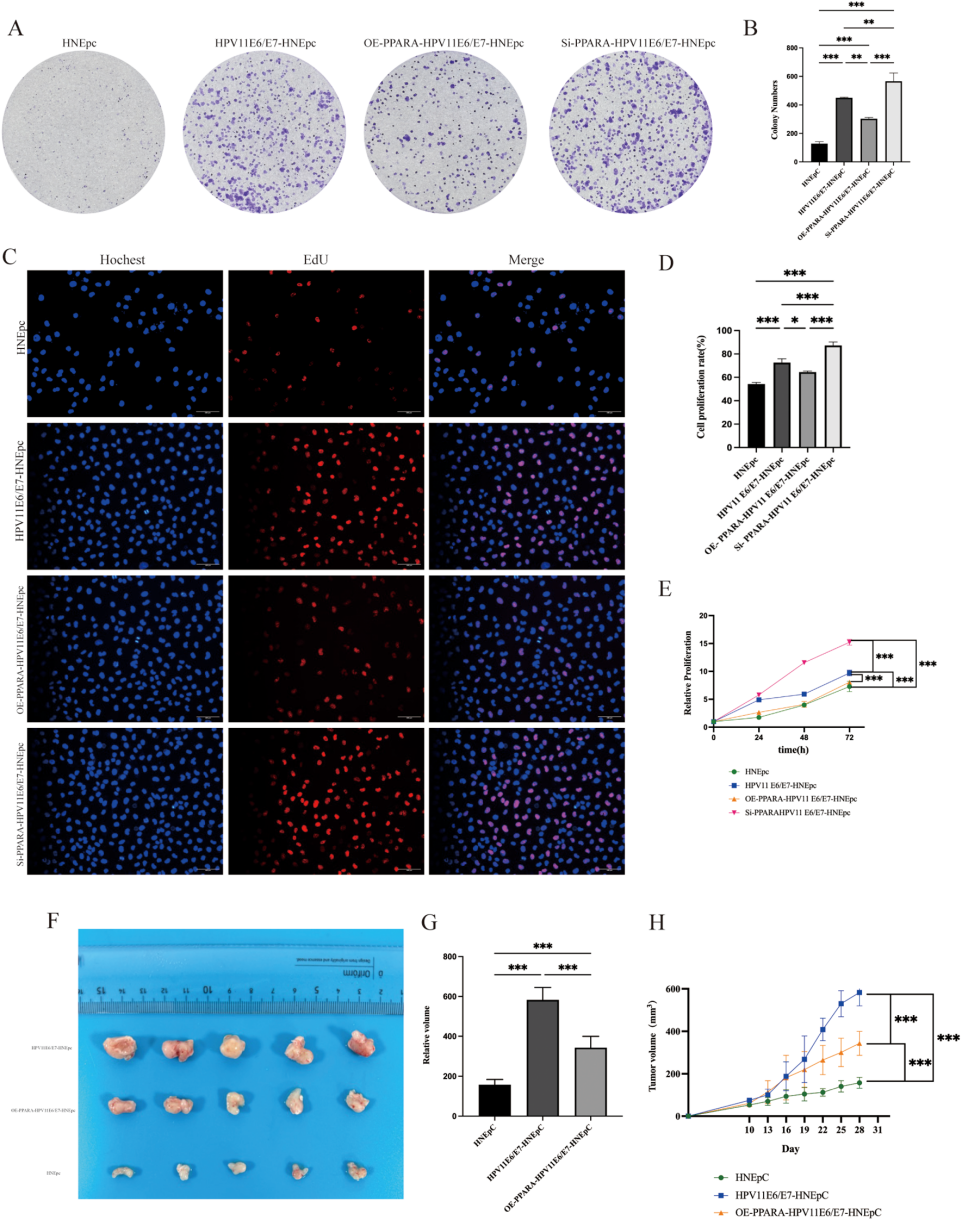
Overexpression of HPV11E6/E7 promoted HNEpC cell proliferation, while *PPARA* overexpression suppressed and *PPARA* knockdown enhanced this effect. **(A, B)** Colony formation assay. **(C, D)** EdU incorporation assay. **(E)** CCK-8 viability assay. **(F, G)** Volume of subcutaneous tumors in nude mice. **(H)** Tumor growth curves. Data are presented as mean ± SD; ***P < 0.001, **P < 0.01, *P < 0.05, ns: not significant.

*In vitro* cell experiments, including CCK-8 assay, plate cloning formation assay, and EdU assay, were performed to evaluate the proliferative capacity of each cell group (**Figures 2A–E**). The results indicated that overexpression of HPV11E6/E7 enhanced the proliferation of HNEpC cells (P< 0.05). Conversely, overexpression of PPARA suppressed this HPV11E6/E7-induced proliferation, whereas knockdown of PPARA further promoted it (P< 0.05).

*In vivo*, subcutaneous tumor xenograft experiments in nude mice demonstrated that HPV11E6/E7-positive cells exhibited accelerated tumor growth (P< 0.001). Notably, overexpression of the PPARA gene significantly inhibited tumor growth under these conditions (**Figures 2F–H**).

### PPARA modulates HPV11E6/E7-induced migration and EMT in HNEpC cells

**Figure 3:**
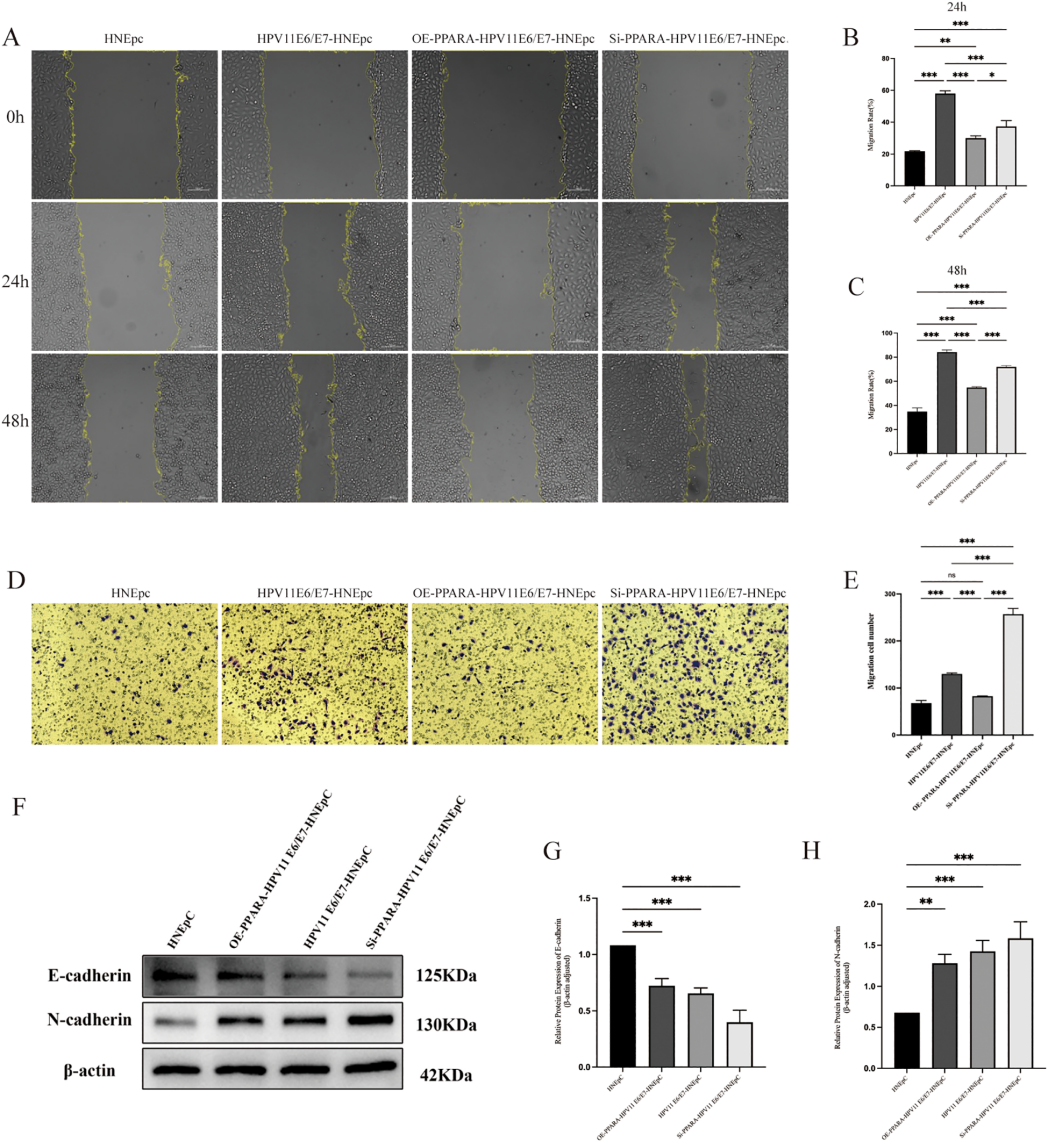
HPV11E6/E7 overexpression promoted HNEpC cell migration and epithelial–mesenchymal transition (EMT); *PPARA* overexpression inhibited, while *PPARA* knockdown enhanced, these effects. **(A)** Wound healing assay. **(B, C)** Quantification of wound closure at 24 h and 48 (h) **(D, E)** Transwell migration assay. **(F–H)** Western blot analysis of E-cadherin and N-cadherin expression. Data are presented as mean ± SD; ***P < 0.001, **P < 0.01, *P < 0.05, ns: not significant.

Wound healing assays and Transwell assays demonstrated that overexpression of HPV11 E6/E7 proteins enhanced cellular migration capacity (P< 0.05). Conversely, overexpression of PPARA suppressed cell migration, whereas knockdown of PPARA facilitated it (**Figures 3A–E**). Consistent with these functional observations, Western blot analysis demonstrated that HPV11E6/E7 overexpression downregulated the epithelial marker E-cadherin and upregulated the mesenchymal marker N-cadherin (P< 0.01). In contrast, PPARA overexpression increased E-cadherin expression and decreased N-cadherin expression, indicating a reversal of the HPV-induced mesenchymal phenotype (**Figures 3F–H**).

### PPARA mediates inhibition of HPV11E6/E7-driven proliferation via autophagy induction

**Figure 4:**
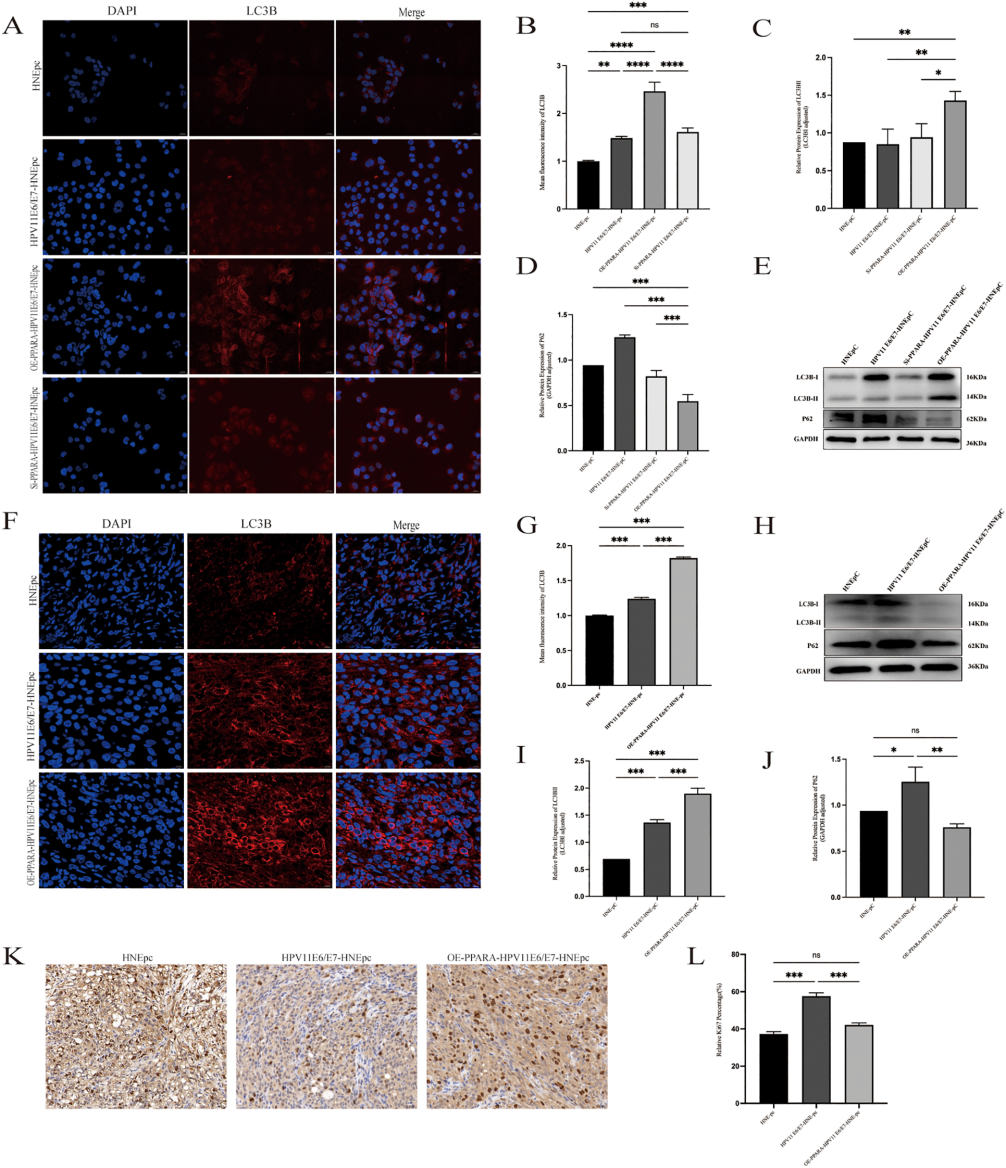
*PPARA* overexpression inhibited HPV11E6/E7-induced proliferation by promoting autophagy. **(A, B)** Immunofluorescence staining and quantification of LC3B puncta. **(C–E)** Western blot analysis of LC3B and p62. **(F, G)** Tissue immunofluorescence of LC3B and quantification. **(H–J)** Western blot of LC3B and p62 in tumor tissues. **(K, L)** Immunohistochemistry and quantification of Ki67 in xenograft tumors. Data are presented as mean ± SD; ***P < 0.001, **P < 0.01, *P < 0.05, ns: not significant.

To investigate the effect of interfering PPARA after HPV11E6/E7 overexpression on cell autophagy in HPV-positive nasal mucosa model. Western blot results demonstrated that PPARA overexpression significantly promoted the expression of autophagy protein LC3BII while markedly reducing autophagy protein P62 expression (**Figures 4C–E**). Immunofluorescence results were similar, with increased fluorescence of the autophagy marker LC3B after overexpression of PPARA (**Figures 4A, B**).

Immunofluorescence (IF) staining and semi-quantitative analysis were performed on excised subcutaneous tumor tissues from nude mouse models (**Figures 4F, G**). Immunofluorescence analysis revealed enhanced LC3B spot fluorescence intensity following PPARA overexpression (P<0.001), indicating increased autophagic flux. Western blot analysis evaluated the protein levels of autophagy markers LC3B and P62 in tumor samples (**Figures 4H–J**). PPARA overexpression promoted LC3B-II expression while decreasing P62 expression (P<0.05), further confirming autophagy activation. Additionally, immunohistochemistry (IHC) detected Ki67 expression (**Figures 4K, L**), revealing significantly reduced Ki67 expression in the PPARA-overexpressing group compared to the HPV11E6/E7 group (P<0.001).

### 3-MA reverses the tumor-suppressive functions of PPARA in HPV11E6/E7-expressing cells

**Figure 5:**
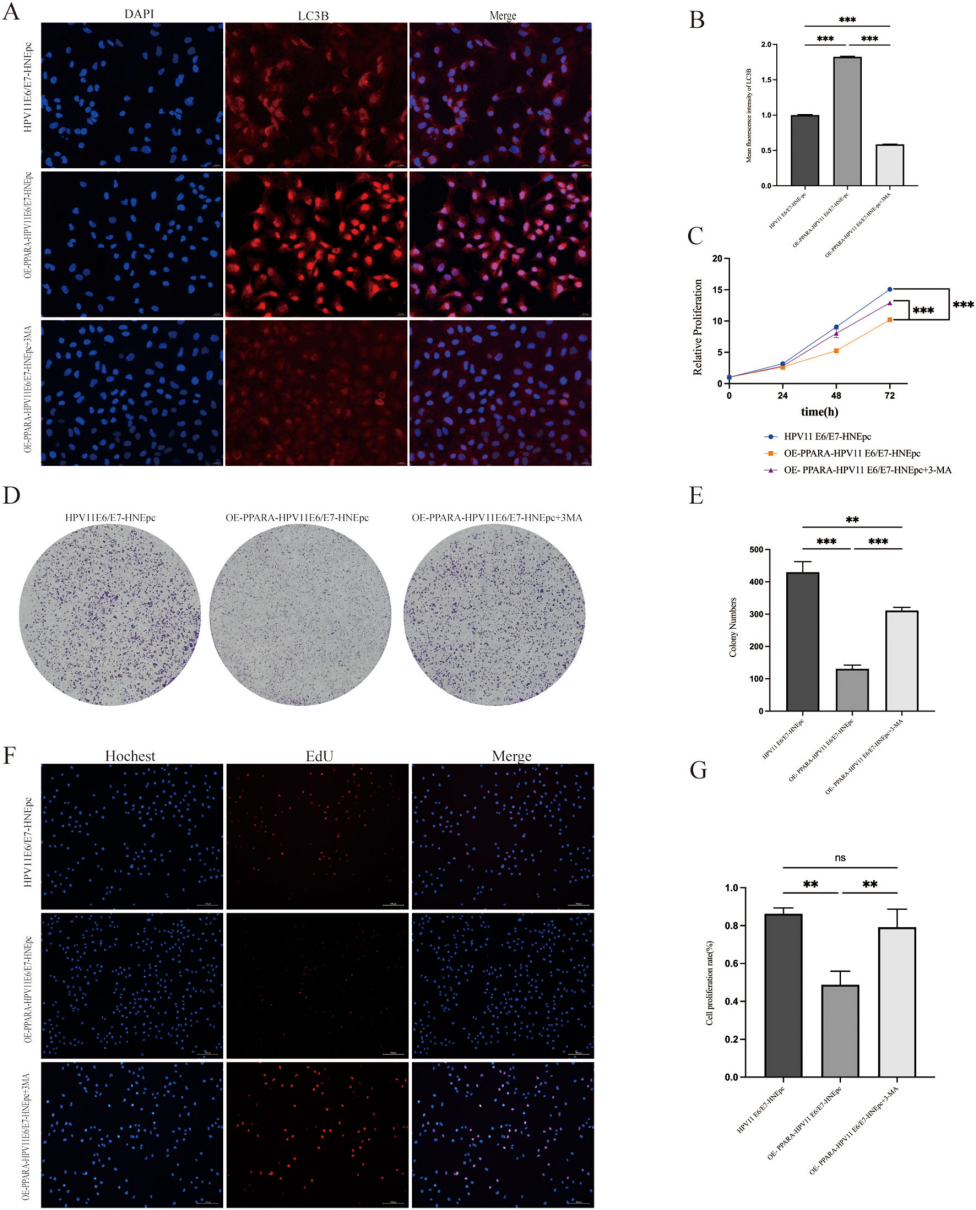
Autophagy inhibitor 3-MA reversed the antitumor effects of *PPARA* overexpression. **(A, B)** LC3B immunofluorescence and quantification after 3-MA treatment. **(C)** CCK-8 assay after 3-MA treatment. **(D, E)** Colony formation assay with 3-MA treatment. **(F, G)** EdU assay with 3-MA treatment. Data are presented as mean ± SD; *P < 0.001, P < 0.01, ns: not significant.

Immunofluorescence analysis revealed significant differences in LC3B fluorescence intensity among the three cell groups (P<0.001). Following 3-MA treatment, the fluorescence signal of the autophagy marker LC3B markedly decreased (**Figures 5A, B**), indicating impaired autophagy activity.

**Figure 6:**
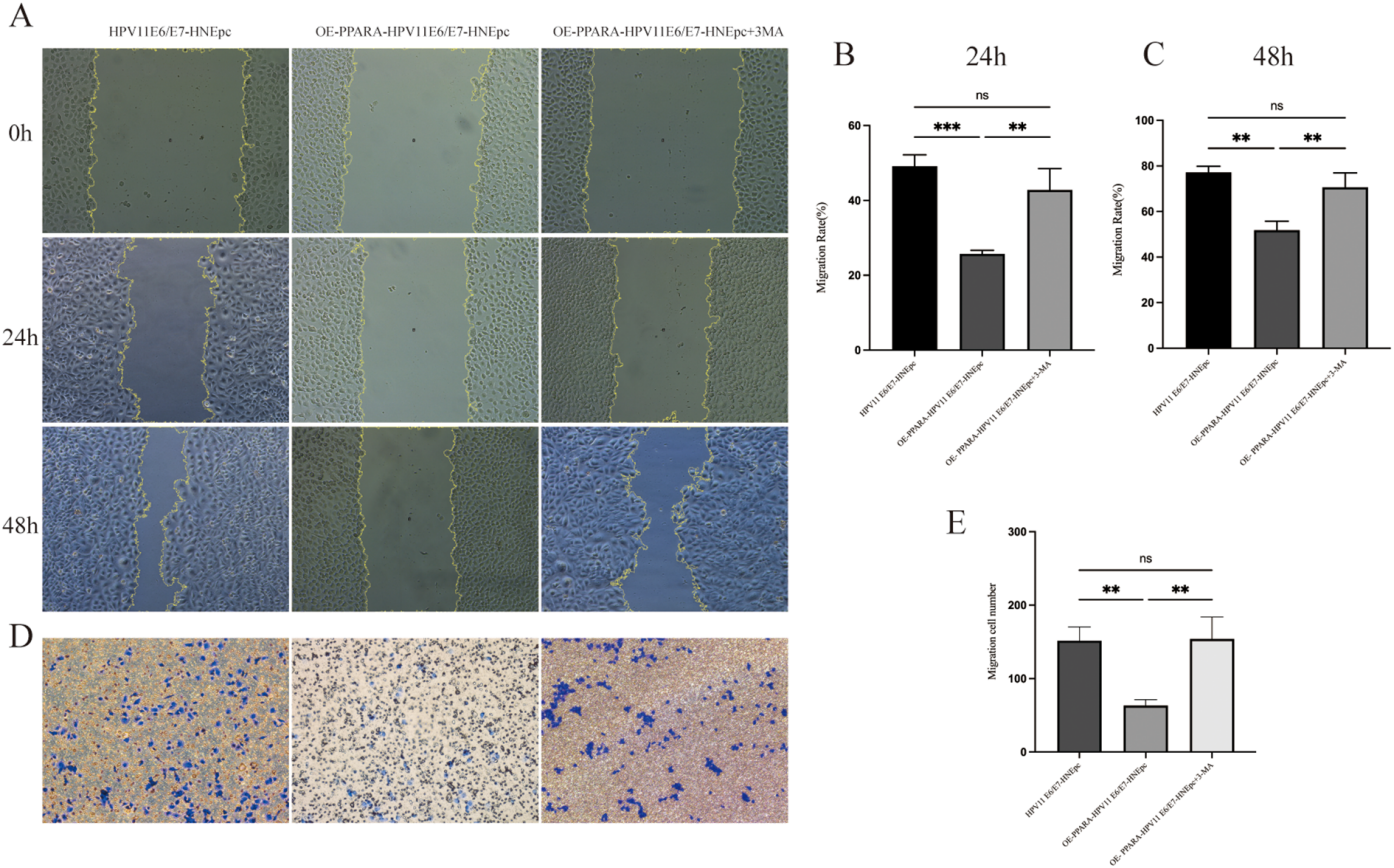
3-MA treatment reversed the inhibitory effect of *PPARA* overexpression on HPV11E6/E7-induced migration. **(A)** Wound healing assay with 3-MA treatment. **(B, C)** Quantification of migration at 24 h and 48 h. **(D, E)** Transwell assay with 3-MA Treatment. Data are presented as mean ± SD; *P < 0.001, P < 0.01, ns: not significant.

*In vitro* functional assays including CCK-8, colony formation, and EdU experiments assessed cell proliferation capacity (**Figures 5C–G**). Results showed that upon 3-MA addition, the antiproliferative effect of PPARA overexpression against HPV11E6/E7-induced HNEpC cells was largely abolished (P<0.01). Similarly, Transwell migration assays demonstrated that 3-MA reversed the inhibitory effect of PPARA overexpression on cell migration (P<0.01). Wound healing assays further confirmed that the PPARA-mediated suppression of cell migration was also abolished in the presence of 3-MA (**Figures 6A–E**).

## Discussion

Numerous domestic and international studies have detected human papillomavirus (HPV) in nasal inverted papilloma (NIP) specimens, supporting a close association between HPV infection and the development of NIP. However, detection rates vary considerably due to factors such as sampling techniques, detection methods, and geographical regions. Although the etiology and pathogenesis of NIP have not been fully elucidated, the role of HPV in head and neck tumors remains a focus of ongoing research. Syrjänen et al. (1987) first identified HPV-DNA in NIP using *in situ* hybridization. Subsequently, multiple studies have explored the relationship between HPV and NIP. Beck et al. (1995) reported a 62.5% HPV positivity rate by PCR among 32 NIP specimens, further supporting an association between HPV infection and NIP. In 1996, Shen et al. (14) demonstrated that HPV contributes to the development of NIP and suggested that different viral types (e.g., HPV6 and HPV11) may exhibit tissue-specific variations. Zhao et al. (15) performed a meta-analysis confirming that HPV infection increases the risk of NIP and may play a critical role in its recurrence. Similarly, Wang et al. (16) used meta-analysis to reinforce the correlation between HPV infection and the occurrence, recurrence, and malignant transformation of NIP.

Our previous study revealed that HPV was detected in 64.36% of NIP tissue samples, with low-risk subtype HPV11 being the most prevalent (32.99%). These findings support the hypothesis that HPV infection promotes NIP development. We further discovered that HPV11 drives proliferation of nasal mucosal cells and modulates M1 macrophage polarization via KDM4A, potentially contributing to the pathogenesis of NIP (17). Characterizing the distribution of HPV subtypes in NIP may aid in predicting clinical behavior and tumor progression. HPV’s carcinogenic potential primarily relies on the sustained expression and activity of the oncogenic E6 and E7 proteins. These proteins, acting as tumor-associated antigens (TAAs), interact with over 100 cellular proteins to collectively alter key cellular processes and promote tumorigenesis (18). E6 and E7 suppress apoptosis and disrupt cell cycle regulation by inactivating the tumor suppressor proteins p53 and pRb. Uncontrolled E6/E7 expression mimics the loss of p53 and pRb function, leading to genomic instability, cellular immortalization, and mutation accumulation, ultimately triggering malignant transformation.

In the clinical data of this study, the majority of NIP patients tested positive for HPV11, and high-throughput sequencing revealed elevated PPARA expression in HPV11-positive tissues. As a nuclear receptor, PPARA serves as a key regulator of cellular autophagy (19). However, the role of autophagy in disease is not simply “promoting” or “inhibiting,” but highly dependent on the specific pathophysiological context. The activating function of autophagy in tumors is influenced by tumor stage and type: during precancerous lesions, autophagy aids in clearing damaged proteins and organelles such as mitochondria, typically exerting tumor-suppressing effects at this stage (20). However, in advanced or metastatic tumors, cancer cells utilize autophagy to acquire nutrients and energy under metabolic and oxidative stress conditions, thereby promoting survival (21).

In the unique clinical context of NIP—a benign proliferative lesion characterized by persistent low-risk HPV infection and chronic inflammation—autophagy may play a more complex and dynamic dual role. On one hand, as demonstrated by the primary findings of this study, the autophagy pathway activated by PPARA indeed exhibits a function of limiting abnormal proliferation and migration of epithelial cells driven by HPV11 E6/E7, consistent with a protective mechanism that maintains tissue homeostasis and prevents excessive hyperplasia. On the other hand, within the persistent inflammatory microenvironment of NIP, autophagy may also be activated as an adaptive cellular response. This helps infected or proliferating cells cope with inflammatory stress and clear toxic substances, thereby promoting their survival in an unfavorable environment and the persistence of the lesion (22).

Therefore, in NIP, the ultimate effect of autophagy may not be simply “tumor suppression” or “tumor promotion,” but rather the manifestation of its fundamental role in maintaining intracellular homeostasis under specific pathological conditions. Our research indicates that autophagy regulated by PPARA primarily antagonizes the virus-promoted proliferation effect in the context of HPV11 infection. This finding underscores the necessity of comprehensively considering specific disease types (benign/malignant), driving factors (viral/inflammatory), and the cellular microenvironment when discussing autophagy functions.

This study has several limitations. First, the experimental model relies on the stable overexpression of HPV11 E6 and E7 oncoproteins, failing to fully replicate the complex biological processes leading to NIP following natural HPV infection. This model primarily captures the sustained tumorigenic drive triggered by viral integration—specifically, the phase of dysregulated E6/E7 expression. Future studies employing HPV11 pseudovirus infection models or organoid epithelial cell cultures will help validate these findings in more physiologically relevant contexts. Secondly, while the subcutaneous xenograft model used here validated the cell-autonomous tumorigenic function of the HPV11-PPARA-autophagy axis, it failed to fully recreate the complex tissue-specific microenvironment of the primary sinus site. Factors such as nasal mucosal-stromal interactions, local immune responses, and anatomical constraints were not incorporated into the model. Our *in vivo* studies primarily elucidate the functional role of this pathway in promoting tumor growth generally. Future studies utilizing *in situ* transplantation models or genetically engineered mouse models capable of inducing sinus lesions would provide valuable validation of this pathway’s relevance within the authentic pathophysiological context of NIP. Thirdly, the mechanism elucidated in this study is primarily based on a single immortalized nasal epithelial cell line model. Whilst the internal chain of evidence within this model is reliable, further validation is required to determine whether its findings can be extrapolated to other cell types (such as primary cells) or *in vivo* settings. Subsequent research will validate this pathway using primary nasal epithelial cells and other HPV-infected cell models associated with head and neck tumors, thereby assessing its broader applicability.

## Conclusion

In summary, this study demonstrates that low-risk HPV11 targets PPARA to influence proliferation and migration in nasal epithelial cells. Overexpression of PPARA promotes autophagy and modulates the local tumor microenvironment, whereas knockdown of PPARA enhances HPV11-induced cellular proliferation and migration. These findings suggest that HPV11 infection contributes to the development and recurrence of NIP, and that targeted upregulation of PPARA may represent a promising therapeutic strategy for HPV-positive NIP.

## Data Availability

The original contributions presented in the study are included in the article/**Supplementary Material**. Further inquiries can be directed to the corresponding author.
