# Supplementary material for: HPV11 targeting PPARA regulates the autophagy to inhibit the occurrence and development of nasal inverted papilloma
Source: Front Oncol. 2026 Jan 28;15:1743808. doi: 10.3389/fonc.2025.1743808 (PMC12890690; doi:10.3389/fonc.2025.1743808)
Supplement: Supplementary file 1 [file DataSheet1.pdf]

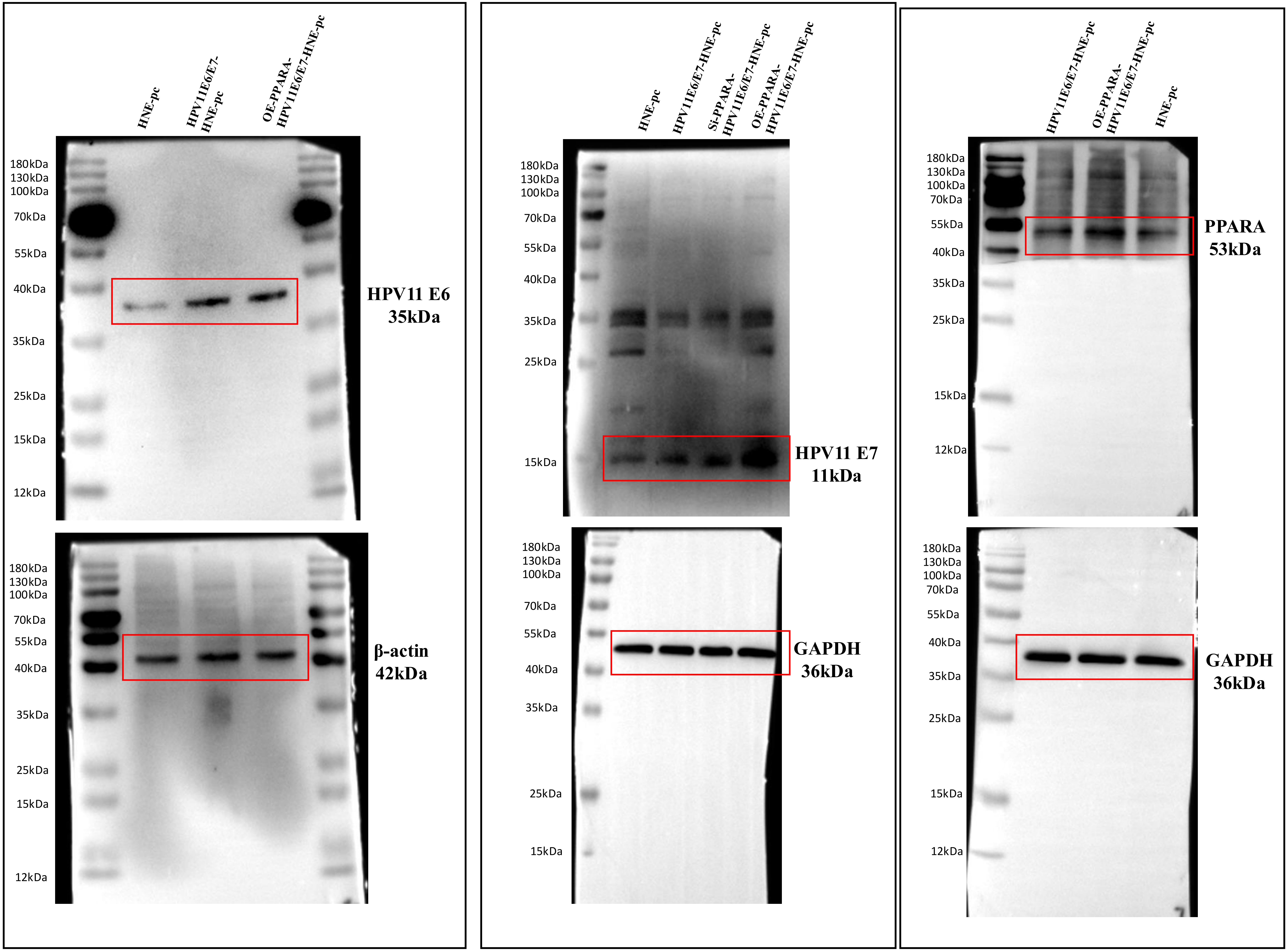
HNE-pc
HPV11E6/E7-HNE-pc
OE-PPARA-HPV11E6/E7-HNE-pc
180kDa
130kDa
100kDa
70kDa
55kDa
40kDa
35kDa
25kDa
15kDa
12kDa
HPV11 E6
35kDa
β-actin
42kDa
HNE-pc
HPV11E6/E7-HNE-pc
Si-PPARA-HPV11E6/E7-HNE-pc
OE-PPARA-HPV11E6/E7-HNE-pc
HPV11 E7
11kDa
GAPDH
36kDa
HPV11E6/E7-HNE-pc
OE-PPARA-HPV11E6/E7-HNE-pc
HNE-pc
PPARA
53kDa
GAPDH
36kDa

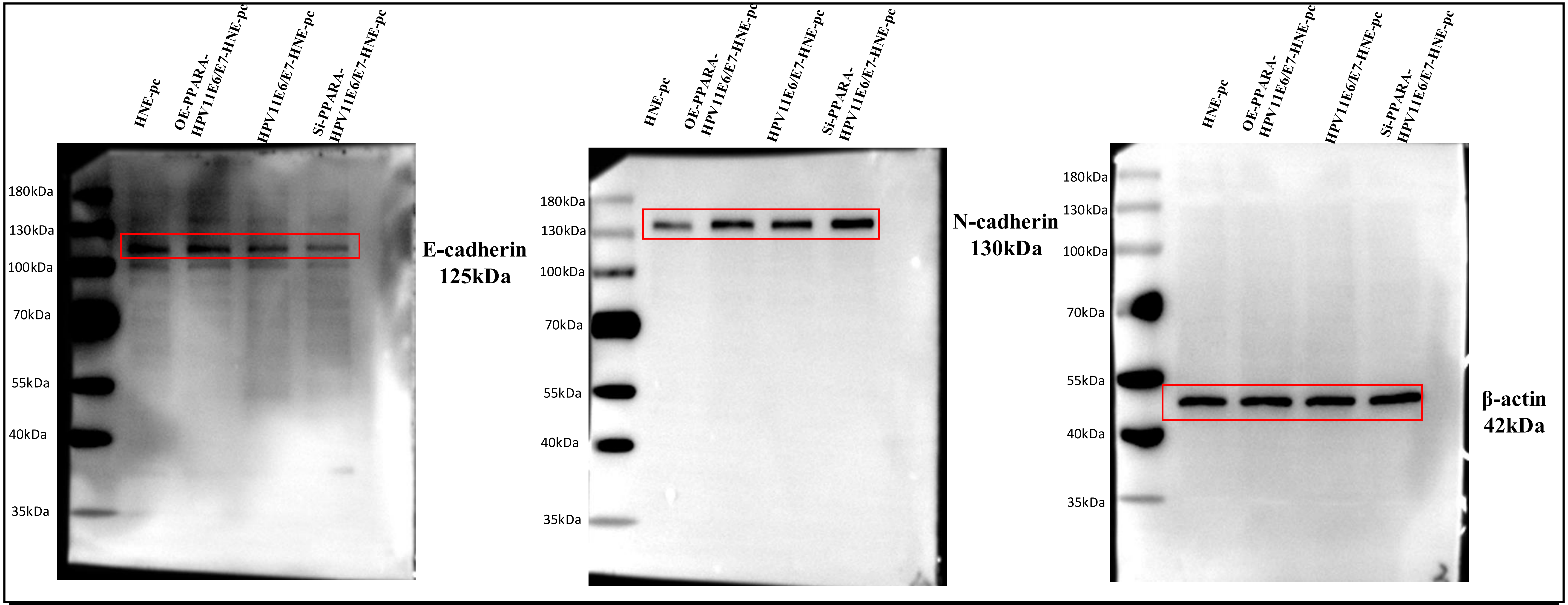
HNE-pc
OE-PPARA-HPV11E6/E7-HNE-pc
HPV11E6/E7-HNE-pc
Si-PPARA-HPV11E6/E7-HNE-pc
180kDa
130kDa
100kDa
70kDa
55kDa
40kDa
35kDa
E-cadherin 125kDa
N-cadherin 130kDa
β-actin 42kDa

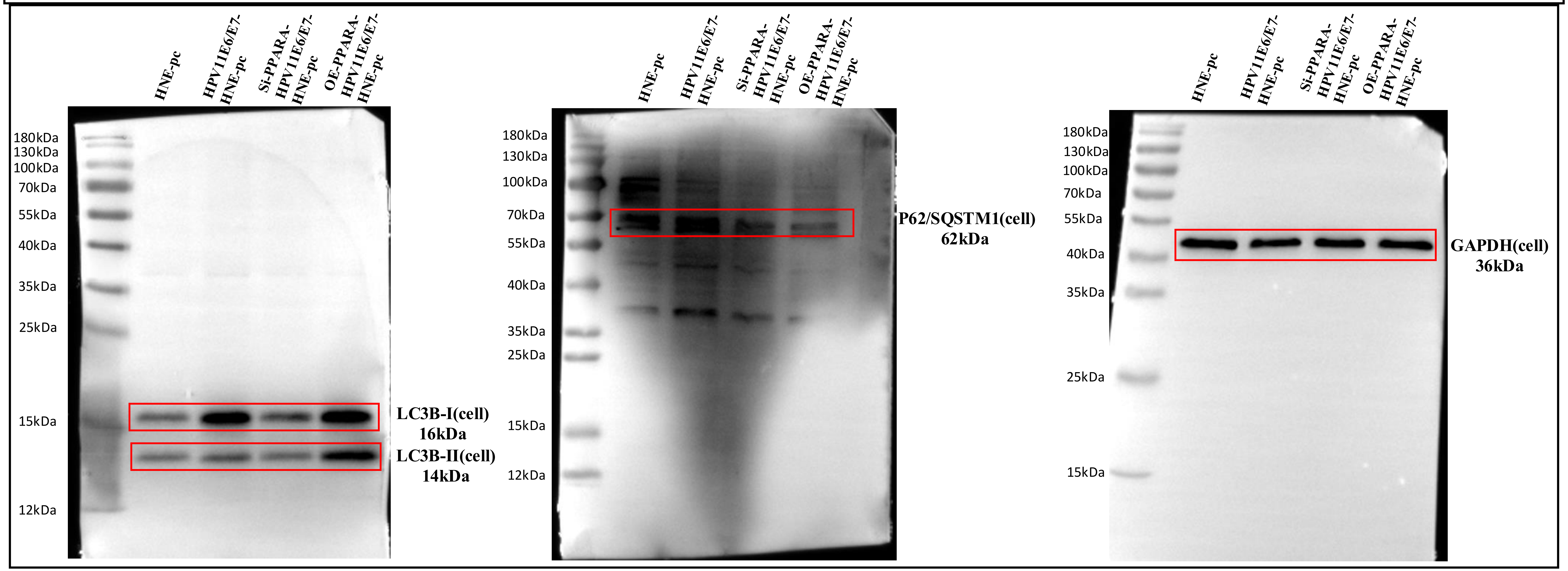
HNE-pc
HPV11E6/E7-HNE-pc
Si-PPARA-HPV11E6/E7-HNE-pc
OE-PPARA-HPV11E6/E7-HNE-pc
180kDa
130kDa
100kDa
70kDa
55kDa
40kDa
35kDa
25kDa
15kDa
12kDa
LC3B-I(cell) 16kDa
LC3B-II(cell) 14kDa
P62/SQSTM1(cell) 62kDa
GAPDH(cell) 36kDa

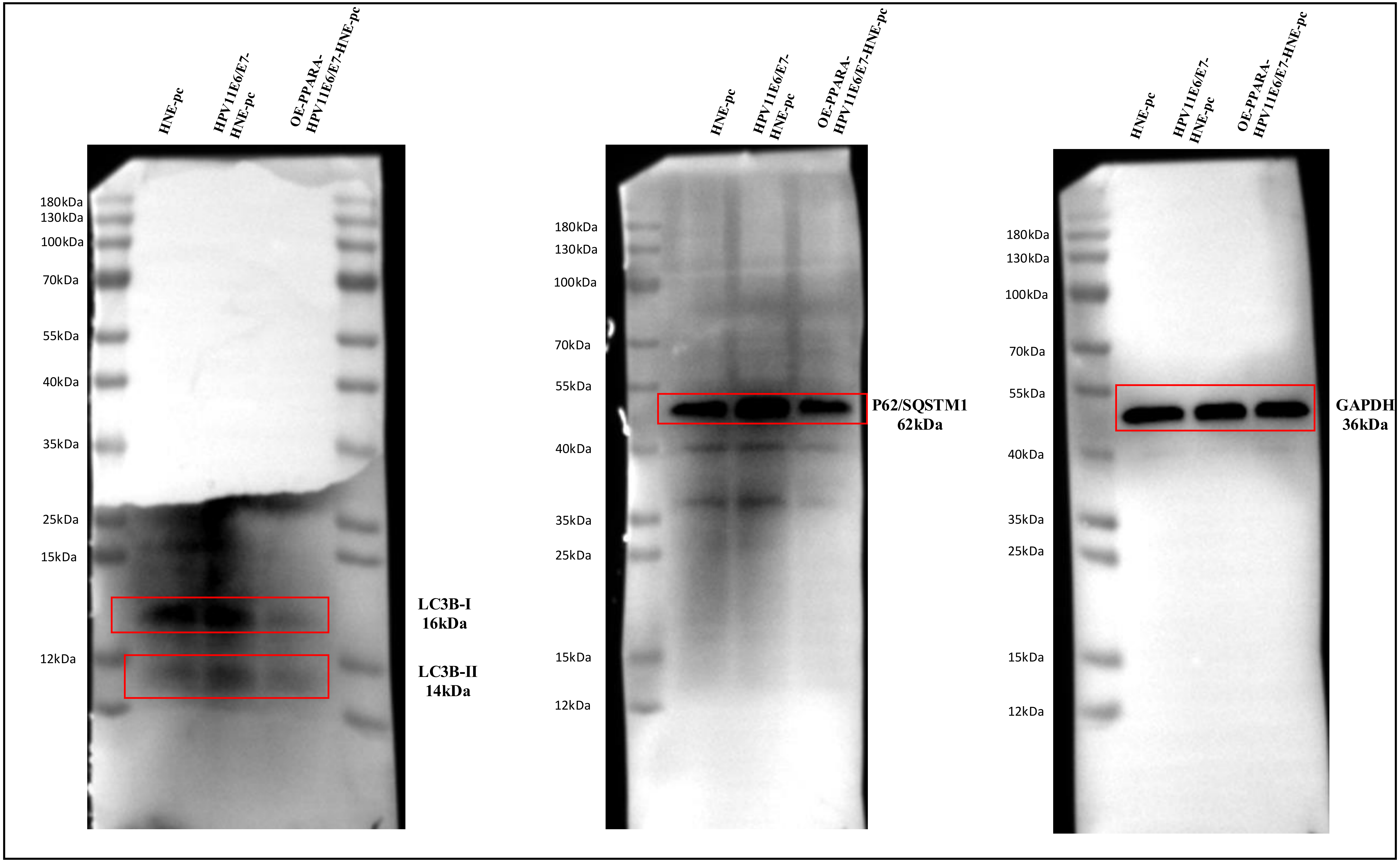
HNE-pc
HPV11E6/E7-HNE-pc
OE-PPARA-HPV11E6/E7-HNE-pc
180kDa
130kDa
100kDa
70kDa
55kDa
40kDa
35kDa
25kDa
15kDa
12kDa
LC3B-I 16kDa
LC3B-II 14kDa
P62/SQSTM1 62kDa
GAPDH 36kDa
