# Supplementary material for: HPV11 targeting PPARA regulates the autophagy to inhibit the occurrence and development of nasal inverted papilloma
Source: Front Oncol. 2026 Jan 28;15:1743808. doi: 10.3389/fonc.2025.1743808 (PMC12890690; doi:10.3389/fonc.2025.1743808)
Supplement: Supplementary file 2 [file DataSheet2.pdf]

Preliminary Findings on HPV and EBV Infection Sites Integrated into Human Chromosomes in Nasal Hyperplastic Diseases

(1) High-throughput sequencing analysis

We employed sixteen viral sequences alongside the human genome reference (hg19) as the reference sequence. The sixteen viral sequences comprised thirteen HPV genomic sequences and two EBV genomic sequences, supplemented by an additional EBV genomic sequence provided in the original reference documentation (NC_009334.1, NC_007605.1, NC_001693.1, NC_001690.1, NC_005134.2, NC_001458.1, NC_001457.1, NC_001691.1, NC_001526.4, NC_001354.1, NC_001694.1, NC_001352.1, NC_016157.1, NC_008189.1, NC_008188.1, NC_007065.1). Matching reads obtained via high-throughput sequencing yielded the following results: The eight samples exhibited read recovery rates ranging from 53% to 84%, with coverage of hg19 varying between 83% and 92%. Sequencing depth spanned 6.43X to 27.67X, while viral coverage (including HPV/EBV) ranged from 0% to 67% with sequencing depths from 0X to 4.33X. Detailed statistical results are presented in Table 1.

Table 1 Statistical Results for 8 Samples

Sample	Number of reads contained in the original file	Number of reads after quality filtering (as a proportion of the original read count)	Total number of replies to the sample	Reply rate	Coverage (hg19)	Sequencing depth (hg19)
15-3006	405287072*2	381612862*2（94%）	767652121	69%	92%	27.67X
15-2135	406510774*2	353688281*2（87%）	800726854	63%	92%	27.14X
14-8438	370618697*2	340167993*2（92%）	699288900	71%	83%	25.73X
14-594	325048325*2	297885513*2（92%）	684180597	53%	84%	18.69X
15-9346	324862648*2	292731834*2（90%）	640647187	68%	92%	23.43X
15-1475	95145152*2	88240263*2（93%）	177404611	84%	91%	7.84X
16-11164	86096133*2	78773508*2（91%）	158826377	80%	91%	6.69X
14-4581	85320797*2	76266022*2（89%）	154221286	78%	89%	6.43X
平均	262361234	238670784	510368492	71%	89%	17.95X

Sample	Both hg19 and HPV+EBV/HPV/EBV (lines)	Only posts tagged with HPV+EBV/HPV/EBV (posts)	Coverage rate (HPV/EBV)	Sequencing depth (HPV/EBV)
15-3006	792/127/665	4875/162/4713	15%/67%	0.69X/2.96X

15-2135	869/88/781	976/51/925	6%/3%	0.19X/0.13X
14-8438	787/58/729	6942/172/6770	5%/49%	0.48X/4.33X
14-594	465/88/377	1048/135/913	10%/3%	0.44X/0.12X
15-9346	2180/206/1974	5126/336/4790	16%/6%	1.06X/0.63X
15-1475	133/0/133	30/0/30	0/1%	0/0.02X
16-11164	264/17/247	350/34/316	2%/2%	0.09X/0.04X
14-4581	226/22/204	1914/24/1890	3%/58%	0.09X/1.31X
平均	725/78/638	2657/114/2543	7%/23%	0.38X/9.54X

Notes:
1. All results were obtained from BWA alignment against hg19 plus viral sequences, comprising 13 HPV and 3 EBV sequences;
2. Column D's quality filtering involved first removing adapters using cutadapt, followed by eliminating low-quality sequences via sickle;
3. Sequencing coverage was calculated using samtools depth as the total number of sites mapped to the reference genome divided by the average length of the reference genome;
4. Sequencing depth was calculated using samtools depth as the total length of reads mapped to the reference genome divided by the average length of the reference genome.

(2) Analysis of HPV Integration Sites in Human Chromosomes

① In HPV(+) specimens, we detected numerous integration sites. Reads that aligned to both HPV and hg19 were selected. Based on their chromosomal positioning, sites with higher expression levels were chosen, with read counts scaled using a logarithmic scale to base 2 (as shown in Table 2).

Table 2 HPV(+) Integration Site Analysis

chr	chr position	HPV hit reads number	Column C: Logarithm to base 2
chrX	26686150	38	5.247927513
chr6	8627112	31	4.95419631
chr4	134407463	23	4.523561956
chr12	4751181	20	4.321928095
chr4	112022661	15	3.906890596
chr1	44123816	12	3.584962501
chr1	29475363	11	3.459431619
chr6	44244115	10	3.321928095
chr1	72652513	8	3
chr15	91575322	8	3
chr16	48376801	8	3
chr21	34169071	8	3
chr7	20619195	8	3

② Based on the aforementioned statistical analysis results, following HPV integration detection, we annotated integration sites using human genomic information and retained those located within exonic regions. Finally, a circular diagram was generated for each patient, synthesising all HPV integration analysis position data to produce a pupil map of viral gene integration sites within the human chromosomal genome for HPV-positive specimens. This constitutes the HPV chromosomal localisation expression map for the leading chromosomes.

High-frequency integration sites of HPV infection with human chromosomes in nasal inverted papilloma

Our research group has preliminarily identified high-frequency integration sites of HPV infection with human chromosomes KDM4A, SRSF4, LONP2, and PPARA in NIP tissue.

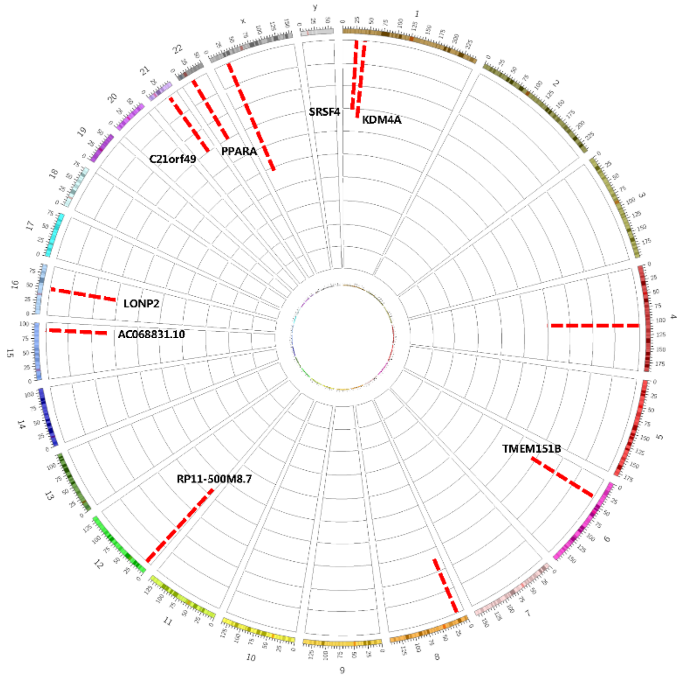

Pupillary map of integration sites for HPV (+) viral genes within the human chromosomal genome in NIP
