# Supplementary figures and images for: HPV11 targeting PPARA regulates the autophagy to inhibit the occurrence and development of nasal inverted papilloma
Source: Front Oncol. 2026 Jan 28;15:1743808. doi: 10.3389/fonc.2025.1743808 (PMC12890690; doi:10.3389/fonc.2025.1743808)

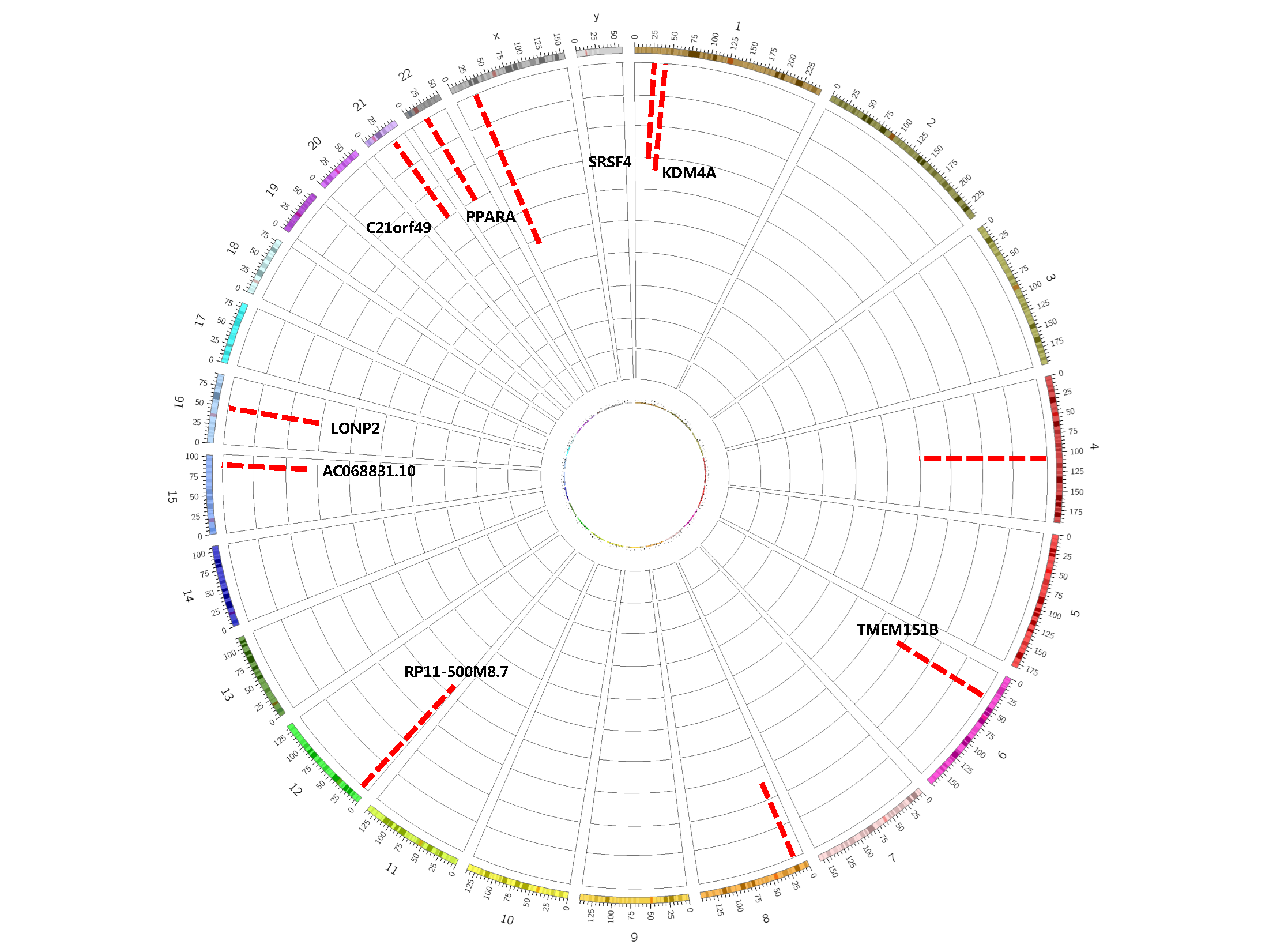

Supplement: Supplementary file 3 [file Image1.png]
